# Editorial: Comprehensive insights into mitral valve prolapse: from biology to future perspectives of treatment passing through diagnostic tools, surgical techniques, and transcatheter options

**DOI:** 10.3389/fcvm.2023.1267418

**Published:** 2023-08-09

**Authors:** Claudia Maria Loardi, Marco Zanobini, Christophe Tribouilloy

**Affiliations:** ^1^ Department of Cardiac Surgery, Tours University Hospital, Tours, France; ^2^ Department of Cardiovascular Surgery, Centro Cardiologico Monzino IRCCS, Milan, Italy; ^3^ Department of Cardiology, Amiens University Hospital, Amiens, France

**Keywords:** mitral valve, mitral valve prolapse, echocardiography, mitral valve repair, mitral annular disjunction, cardiac magnetic resonance

**Editorial on the Research Topic** Comprehensive insights into mitral valve prolapse: from biology to future perspectives of treatment, passing through diagnostic tools, surgical techniques and transcatheter options

Mitral valve prolapse (MVP) affects 2%–3% of the population and represents the first cause of primary chronic mitral regurgitation (MR) (1). Although defined as “an abnormal systolic protrusion of mitral valve leaflets in the left atrium”, MVP includes a wide spectrum of anatomical conditions, ranging from Barlow's disease to fibroelastic deficiency (FED). Barlow's disease patients are younger and present redundant myxoid leaflets and elongated (rarely ruptured) chordae, whereas FED is associated with translucent tissue, chordal rupture, and flail leaflet in older patients (2).

Despite thousands of articles on MVP, this pathological entity still presents some blind spots: a full comprehension of its underlying biological bases is far from being achieved (3), as is the rising evidence of a link between MVP, mitral annular disjunction (MAD), and malignant arrhythmias (4).

Given the excellent results of surgical repair (5), indications of treatment are in continuous evolution, aiming at weighing the predicted risk, repair feasibility, and precocious markers of cardiac impairment (6). This trend translates into a parallel effort to combine all the diagnostic tools for detecting, as early as possible, the criteria of MR severity and their possible impact on heart function. If echocardiography represents the first-line imaging modality using qualitative, semiquantitative, and quantitative parameters, cardiac magnetic resonance (CMR) plays an emerging role by showing high accuracy and reproducibility (7).

Concerning therapeutic options, less invasive surgical approaches for mitral valve repair (MVR) (video-assisted and robotic surgery) and transcatheter techniques are spreading while maintaining safety and efficacy (8).

This Special Issue delves into all these significant aspects through eleven articles.

Delwarde et al. reviewed all the current knowledge concerning the genetics of MVP. They distinguished MVP occurring as a part of syndromic conditions (e.g., Marfan syndrome) from the more frequent non-syndromic form, isolated or familial, which can be stratified into Barlow's disease, Filamin A-related MVP, and FED. After summarizing all the genetic defects associated to these conditions, the authors considered a potential genetic link between MVP and ventricular arrhythmias or cardiomyopathy.

The review by Ronco et al. described the altered molecular pathways underlying MVP, including TGF-β overexpression and dysregulated extracellular matrix organization. A special attention was addressed to their experimental response to pharmacological inhibition, thus possibly opening the way for interventional treatment slowing down MVP progression.

Dieterlen et al. conducted a review focused on MVP-induced fibrosis, which is a consequence of increased mechanical stress on the myocardium due to the altered motion of the mitral valve, independently of volume overload. An overview of the histopathological mechanisms and of the limits of current diagnostic tools was conducted, highlighting the impact of regional fibrosis on malignant arrhythmia development and on left ventricular (LV) dysfunction after MVR, a phenomenon whose extent is difficult to predict, especially in patients presenting a normal preoperative contraction.

Concerning this interesting issue of LV systolic dysfunction after MVR, Petolat et al. investigated the prognostic impact of preoperative forward-flow indices. They showed that LVOT_TVI_ represents an independent predictor of postoperative myocardial performance impairment, with the best accuracy obtained with LVOT_TVI_ ≤15 cm. Such a finding may help in deciding the optimal surgery timing and in predisposing an adequate peri-operative inotropic support in high-risk patients.

Mantegazza et al. reviewed the role of three-dimensional echocardiography in all phases of the MVP course: diagnosis, morphological description, quantification of regurgitation, preoperative planning, intraoperative and intraprocedural (in case of percutaneous treatment) guiding, and post-surgery evaluation. By comparing the features of the latest three-dimensional technology with standard trans-thoracic (TTE) and transesophageal echocardiography (TOE), the authors have encouraged its widespread use to further improve patient outcome.

Altes et al. addressed the problem of the accurate quantification of primary MR by TTE and TOE and underlined the role of CMR. Since the existence of multiple echocardiographic parameters to grade MR may result in discrepancies, the authors described the respective advantages and pitfalls of each different quantitative method. They concluded that MR grading classification should ideally be replaced by a “continuum” vision that is able to include the hemodynamic consequences of MR, whose assessment is better appraised by MR regurgitant fraction.

Pace et al. aimed to apply strain echocardiography to stratify the rhythmic risk in MVP patients. As outlined by Kubala et al., “arrhythmic MVP” is an underappreciated cause of sudden cardiac death whose comprehension in terms of prediction, pathophysiology, management, and prognosis is still incomplete (9). In patients presenting with severe ventricular arrhythmias, myocardial deformation analysis identified specific contraction patterns with increased post-systolic index and myocardial dispersion, suggesting the need for the inclusion of these parameters in the global evaluation of degenerative MR.

Vermes et al. reviewed the evolving role of CMR in the global assessment of MVP. If echocardiography represents the gold standard for diagnosis, CMR is a reliable tool to assess MR severity by accurately measuring the regurgitant volume and the regurgitant fraction and to detect LV function, remodeling, and fibrosis. This allows to evaluate not only MVP itself but also its hemodynamic consequences on the cardiac chambers. Moreover, it permits to associate the detection of fibrosis with the possible development of ventricular arrhythmias. The authors strongly have recommended addressing patients for CMR in the case of MR severity discrepancies between echocardiography and clinics or in the case of persistent doubt on MR severity using echocardiography.

Biondi et al. investigated the three-dimensional extension of MAD in patients affected by MVP and its implications for surgical repair. Two major phenotypes of MAD (a bimodal shape and a more uniform distribution) were identified in Barlow patients at TOE. Interestingly, this complex anatomic feature failed to translate in an increased surgical complexity of repair, since basic techniques could be successfully employed in most patients.

The article by Carpenito et al. addressed the long-standing dispute between the promoters of early surgery in MVP and those sustaining a more prudent attitude waiting for the appearance of symptoms, LV dilation, or impairment. The authors described the so-called watchful surgery approach, proposing a sort of guiding algorithm based on the real severity of MR, the true patient's asymptomatic condition, the complete evaluation of the cardiac chambers, and the expected durability of MVR.

Van Kampen et al. presented their experience concerning the evolution of MVR from the traditional sternotomy approach to the recent introduction of the robotic surgery. They have concluded that a dedicated program based on stepwise training allows a gradual transition to robotic surgery without compromising patient safety with improved institutional outcomes, increased MVR volume, and diversification of techniques.

In conclusion, this Special Issue discusses several emerging areas of research of MVP (10), including the understanding of the biologic and genetic mechanisms of prolapse onset and progression, the integration of all the available diagnostic tools, and the prevention of sudden cardiac death. All the advances in these fields are expected to result in an improvement of therapeutic attitudes and, perhaps, in a modification of the surgical and percutaneous indications.
